# Nucleating amoeboid cancer cell motility with Diaphanous related formins

**DOI:** 10.1002/cm.21880

**Published:** 2024-05-18

**Authors:** Neelakshi Kar, Jeremy S. Logue

**Affiliations:** ^1^ Regenerative and Cancer Cell Biology Albany Medical College Albany NY USA

**Keywords:** actin, amoeboid, bleb, cancer, Diaphanous related formin

## Abstract

The tissue invasive capacity of cancer cells is determined by their phenotypic plasticity. For instance, mesenchymal to amoeboid transition has been found to facilitate the passage of cancer cells through confined environments. This phenotypic transition is also heavily regulated by the architecture of the actin cytoskeleton, which may increase myosin contractility and the intracellular pressure that is known to drive bleb formation. In this review, we highlight several Diaphanous related formins (DRFs) that have been found to promote or suppress bleb formation in cancer cells, which is a hallmark of amoeboid migration. Based on the work discussed here, the role of the DRFs in cancer(s) is worthy of further scrutiny in animal models, as they may prove to be therapeutic targets.

## 1 INTRODUCTION

Cell migration is essential during embryogenesis, wound healing, and immune surveillance. In cancer, the migration of cells away from the primary tumor is a key step within the metastatic cascade. To date, the therapeutic targeting or inhibition of cancer cell motility has proved challenging (Coussens et al., 2002; Steeg, 2016). The plasticity of phenotypes by which cells may invade tissues, in particular, endows cells with the ability to effectively evade therapies targeting migration (Logue et al., 2018; Sahai & Marshall, 2003; Tozluoglu et al., 2013; Ullo & Logue, 2018; Wolf et al., 2003). Consequently, the identification of molecular mechanism(s) that drive cancer plasticity is of great interest.

Classically, the assumption of a migratory phenotype follows an epithelial to msenchymal transition. During mesenchymal migration, cells utilize focal adhesions to attach to and sense the mechanical properties of the extracellular matrix (ECM). The leading edge of a mesenchymal cell is advanced by Brownian ratcheting, which facilitates the barbed end elongation of actin filaments against plasma membrane (PM) tension (Pollard & Borisy, 2003). The motive force for cell movement, however, is generated by the coupling of retrograde actomyosin flow to the ECM by focal adhesions (Pollard & Borisy, 2003). To effectively pull themselves through ECM, cells may also require the action of matrix metalloproteases (MMPs) (Yamada & Sixt, 2019). Migration may also proceed by way of a mesenchymal to amoeboid transition (MAT), which has been observed in the absence of MMP activity (Graziani et al., 2022; Wolf et al., 2013).

**FIGURE 1:**
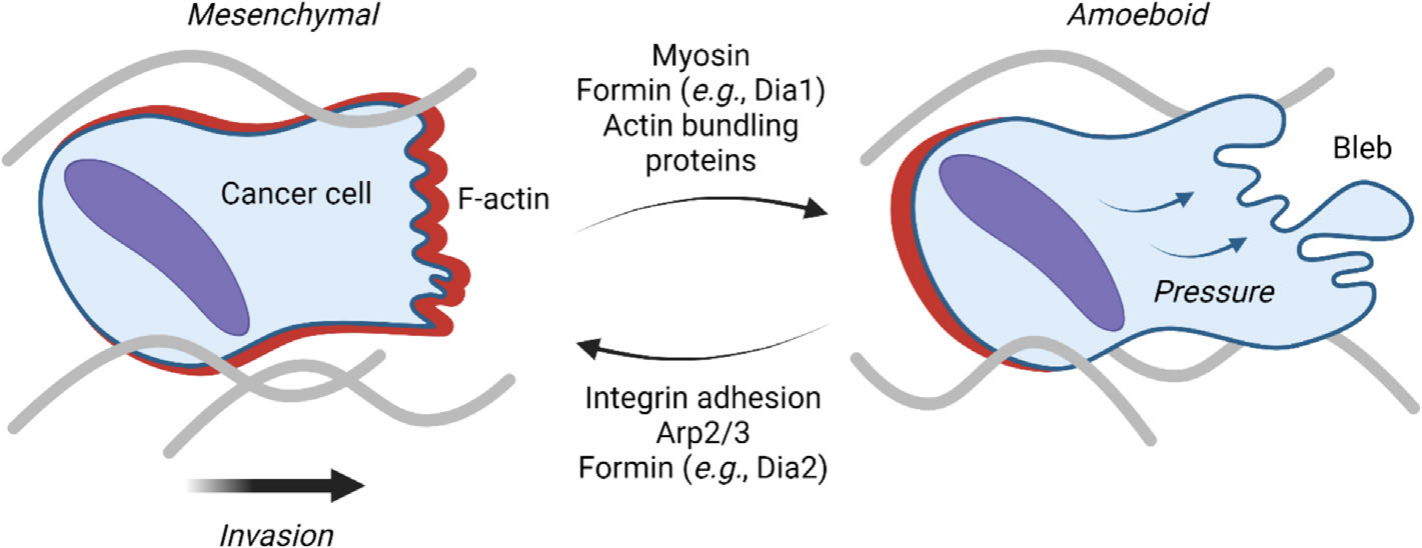
Drivers of mesenchymal versus amoeboid migration in cancer cells. Amoeboid migration is driven by myosin and actin bundling proteins, whereas integrin adhesion and Arp2/3 drive mesenchymal migration. A formin may drive mesenchymal or amoeboid migration. Protrusion of the leading edge is driven by F‐actin in mesenchymal cells, whereas protrusion of the leading edge is driven by intracellular pressure (i.e., blebs) in amoeboid cells. Created with BioRender.com

During amoeboid migration, cells protrude a plasma membrane (PM) bleb. A bleb forms when a segment of the PM separates from the cortical actin cytoskeleton. As the inflation of a bleb requires the flow of cytoplasm, cells with non‐equilibrated hydrostatic pressure are more prone to forming blebs (Charras et al., 2005). Accordingly, cells with a high level of myosin contractility are more likely to display amoeboid features (Figure 1) (Liu et al., 2015; Tinevez et al., 2009). The lifetime of a single bleb is very short, lasting less than a minute (Charras et al., 2008). For cell movement in ECM, blebs may interdigitate between individual collagen fibers and pull a cell forward as they retract (Tozluoglu et al., 2013). Accordingly, cell movement requires the ECM to be sufficiently stiff to resist cell‐generated forces. For the highest rates of cell movement, bleb formation is restricted to the leading edge (Tozluoglu et al., 2013). Polarization is thought to be mediated in part by the Ezrin, Radixin, and Moesin (ERM) family of proteins. By linking the PM to cortical actin cytoskeleton, concentrations of Ezrin can locally suppress the formation of blebs (Lorentzen et al., 2011; Tozluoglu et al., 2013). The ERM family can be activated by phosphorylation downstream of Rho and by acidic phospholipids, including phosphatidylinositol 4,5‐bisphosphate (Neisch & Fehon, 2011). In newly formed blebs, Ezrin is one of the first proteins to be observed at the PM; therefore, Ezrin may play an essential role in orchestrating the re‐assembly of the cortical actin cytoskeleton before myosin recruitment and bleb retraction (Charras et al., 2008). Using mass spectrometry‐based proteomic analyses in melanoma cells, the cortical actin cytoskeleton in blebs has been shown to be composed of >150 proteins (Biro et al., 2013; Bovellan et al., 2014). A few in‐depth studies of specific actin‐binding proteins in blebbing cells have also been conducted.

### 1.1 Leader bleb‐based migration

We previously demonstrated that the actin capping and bundling protein, Epidermal growth factor receptor kinase substrate 8 (Eps8), promotes leader bleb‐based migration (LBBM) (Logue et al., 2015). During LBBM, which has also been called “fast amoeboid migration,” cells migrate in the direction of a large and stable bleb (Liu et al., 2015). Importantly, the formation of a leader bleb is promoted by two‐dimensional or vertical confinement (Liu et al., 2015; Logue et al., 2015; Ruprecht et al., 2015). While the precise mechanism(s) by which cells transition to this phenotype are incompletely described, recent evidence suggests a critical role for Ca^2+^ from the ER and arachidonic acid liberated from a stretched nuclear membrane by cytosolic Phospholipase A2 to upregulate actomyosin contractility (Lomakin et al., 2020; Venturini et al., 2020). The motive force for cell movement is generated by cortical actomyosin flow in leader blebs, which depends on actin turnover at their base by ADF and cofilin‐1 (Bergert et al., 2015; Ullo & Logue, 2021). On the other hand, Eps8 promotes LBBM by bundling actin filaments, which supports myosin contractility. Importantly, the capping activity of Eps8 is suppressed by Erk phosphorylation (Menna et al., 2013). Using atomic force microscopy (AFM) based measurements of cell mechanical properties, the suppression of actin capping was found to make Eps8 a more effective bundler (Logue et al., 2015). Thus, the architecture of the actin network can pivot cells toward an amoeboid phenotype. While the generation of a branched actin network by Arp2/3 is essential for the formation of a lamellipodia, the inhibition of Arp2/3 has been shown to promote blebbing (Logue et al., 2018). This phenomenon can likely be attributed to an enhancement in actomyosin contractility, as branches sterically hinder filament sliding (i.e., contraction) (Muresan et al., 2022). In agreement with this concept, AFM of cells in which Arp2/3 has been inhibited show elevated levels of intracellular pressure (Cartagena‐Rivera et al., 2016). The level of network connectivity and actin filament length also heavily influence contractility, where in each case there is an optimum (Chugh et al., 2017; Ennomani et al., 2016). While linear actin filaments favor actomyosin contractility, it is not always clear how a specific nucleator may affect the balance of mesenchymal vs. amoeboid behavior.

### 1.2 Diaphanous‐related formins

**FIGURE 2:**
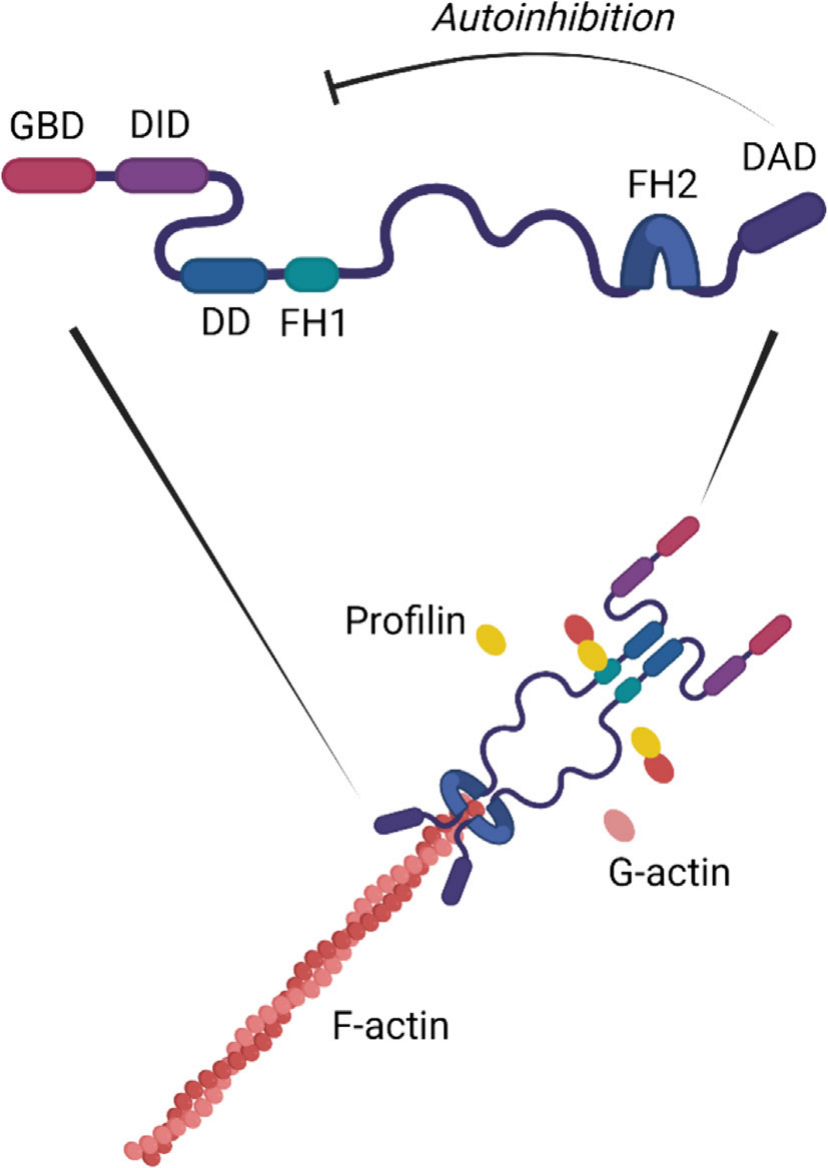
Diaphanous related formins. Upon binding a Rho GTPase, release from autoinhibition induces Diaphanous related formin (DRF) dimerization and barbed‐end binding. Actin polymerization is accelerated by the recruitment of profilin bound G‐actin by the FH1 domains. GTPase binding domain (GBD), Diaphanous inhibitory domain (DID), dimerization domain (DD), formin homology 1 (FH1), formin homology 2 (FH2), Diaphanous‐autoregulatory domain (DAD). Created with BioRender.com

The diaphanous‐related formin (DRF) subfamily of proteins elongate linear actin filaments from free barbed ends (Chesarone et al., 2010). Within a given cell, around half of the globular actin pool is unpolymerized (i.e., G‐actin). Binding of G‐actin to the exchange factor, profilin, not only exchanges ADP for ATP, but also prevents the uncontrolled polymerization of filamentous‐actin (F‐actin) in cells (Kaiser et al., 1999). Elongation factors, such as DRFs, promote elongation by encircling the barbed end with a formin homology 2 (FH2) domain dimer (Chesarone et al., 2010). On the other hand, the proline‐rich formin homology 1 (FH1) domains from each protomer in the dimer recruit G‐actin bound profilin (Figure 2) (Chesarone et al., 2010). In so doing, DRFs in essence increase the local concentration of polymerization competent G‐actin, increasing barbed elongation. Importantly, the activity of DRFs is tightly regulated. Typically, DRFs are maintained in an autoinhibited state by a Diaphanous inhibitory domain and Diaphanous‐autoregulatory domain (DAD) intramolecular interaction (Chesarone et al., 2010). Autoinhibition can be relieved by binding of Rho or cdc42 to the GTPase binding domain (GBD). Consequently, DRFs can be activated downstream of guanine nucleotide exchange factors (GEFs).

### 1.3 Regulation of Dia1 and 2 by DIP

**FIGURE 3:**
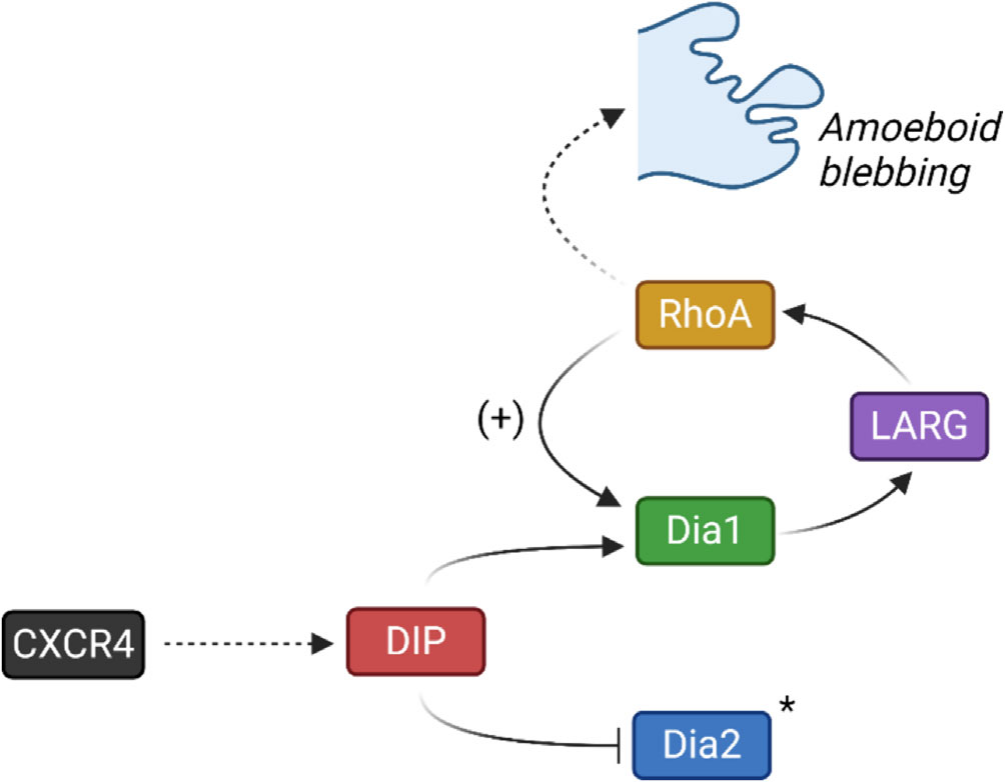
Schematic representation of Dia1 and Dia2 regulation by DIP. Briefly, the activation of Dia1 by DIP starts a positive feedback loop (+) that activates RhoA through LARG. In contrast, Dia2 is inhibited by DIP, which is frequently lost (*) in advanced cancers. Specific chemokines have been shown to induce the binding of DIP to Dia2. Created with BioRender.com

In melanoma, amoeboid migration is promoted by lysophosphatidic acid receptor (LPAR) signaling, which is a G_12/13_ coupled G‐protein coupled receptor (GPCR) (Kitzing et al., 2007). The GEF, Leukemia‐associated Rho guanine nucleotide exchange factor (LARG), is activated by G_12/13_ coupled receptors (Kourlas et al., 2000). In turn, the activation of Rho by LARG activates Diaphanous related formin 1 (Dia1). Interestingly, this sets in motion a positive feedback loop in which Dia1 further activates LARG through direct binding of its FH2 domain (Figure 3) (Kitzing et al., 2007). Consequently, the activation of the LPAR, LARG, and Dia1 signaling pathway promotes the amoeboid invasion of melanoma into hydrogels (Kitzing et al., 2007). On the other hand, Dia2 but not Dia1 is inhibited by Dia interacting protein (DIP) (also known as SPIN90), despite being capable of binding to both Dia1 and Dia2 (Eisenmann et al., 2007). Interestingly, the inhibition of Dia2 by DIP suppresses the formation of filopodia but promotes bleb formation in cervical cancer cells (Table 1) (Eisenmann et al., 2007). In breast cancer cells, C‐X‐C motif chemokine 12 (CXCL12) signaling was found to induce the binding of DIP to Dia2 (Wyse et al., 2012). CXCL12 can be secreted by cancer‐associated fibroblasts in the tumor microenvironment (Hinton et al., 2010; Muller et al., 2001). DIP and Dia2 were found together by high‐resolution imaging at nascent blebs, which suggests that they may work together to initiate bleb formation (Wyse et al., 2012). Thus, these data would suggest that the loss of Dia2 promotes metastasis. Indeed, Dia2 levels have been shown to be frequently downregulated in advanced cancer (Hager et al., 2012). Notably, Dia2 is located on chromosome 13, which is frequently altered in cancer (Hager et al., 2012). In ovarian cancer cells, the loss of Dia2 promotes the invasion of cells from spheroids into collagen gels by inducing amoeboid migration (Pettee et al., 2014). In support of these data, Rho/ROCK signaling was also found to be required for invasion (Pettee et al., 2014). In ovarian cancer cells migrating on glass coverslips, Dia2 could be found in lamellipodia (Pettee et al., 2014). Thus, Dia2 could antagonize amoeboid migration by favoring the formation of lamellipodia over blebs.

**TABLE 1:** DRF driven protrusion types.

Formin	Protrusion type	Cancer type	Key references
Dia1	Bleb	Melanoma	Kitzing et al., 2007
Dia2^a^	Filopodia, lamellipodia	Ovarian, breast, prostate	Eisenmann et al., 2007, Wyse et al., 2012, Hager et al., 2012
FHOD1	Bleb	Ovarian	Hannemann et al., 2008
FHOD3	Filopodia	Ovarian	Paul et al., 2015
FMNL1^b^	Bleb	Chronic myelogenous leukemia	Han et al., 2009
DAAM1	Bleb	Melanoma	Rodriguez‐Hernandez et al., 2020

^a^ Dia2 is frequently lost in advanced cancers.

^b^ Requires myristoylation.

A molecular mechanism for how Dia1 and DIP regulate the cortical actin network has been elucidated. Using electron microscopy, RNAi of Dia1 was found to lead to the formation of large gaps in the cortical actin network (Cao et al., 2020). Upon RNAi of DIP, cortical actin thickness and density were decreased and increased, respectively, which suggests that DIP has a pivotal role in regulating cortical actin nucleation (Cao et al., 2020). DIP is also known to bind Arp2/3, but the physiological significance of this interaction is poorly understood (Kim et al., 2006). Surprisingly, DIP, Arp2/3, and Dia1 have been found together in vitro at the pointed ends of elongating actin filaments (Cao et al., 2020). In this context, Arp2/3 mimics a barbed end. Moreover, DIP competes with WAVE for Arp2/3, which would suggest that DIP has the potential to potentially shift the actin network from a branched to linear configuration. Interestingly, Dia1 has also been found to interact with IQGAP1 (Cao et al., 2020). This interaction may help to target Dia1 to specific subcellular locations. RNAi of IQGAP1 also leads to large gaps, thus it may help to scaffold together several factors important for nucleating a cortical actin network (Cao et al., 2020). Both Dia1 and Arp2/3 have been found in blebs from melanoma cells by mass spectrometry (Biro et al., 2013; Bovellan et al., 2014). In addition to actin, the regulation of microtubules by formins may also promote amoeboid migration in cancer cells.

### 1.4 Regulation of microtubule stability by Dia2

**FIGURE 4:**
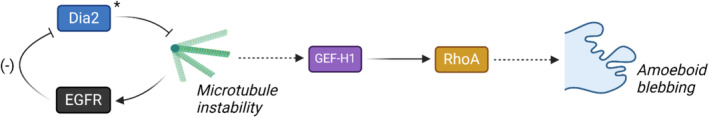
Schematic representation of a Dia2 negative feedback loop. Briefly, the inhibition of Dia2 by DIP starts a negative feedback loop (−) that induces microtubule instability that in turn releases GEF‐H1 to activate RhoA. Dia2 is frequently lost (*) in advanced cancers. Created with BioRender.com

The guanine nucleotide exchange factor H1 (GEF‐H1) is sequestered by microtubules. Accordingly, disruption of the microtubule lattice is known to release GEF‐H1 and upregulate Rho/ROCK signaling (Krendel et al., 2002). Dia2 is known to promote mesenchymal migration, whereas its absence promotes amoeboid migration. This switch in phenotype is associated with de‐stabilization of microtubules, as Dia2 has been shown to stabilize microtubules (Hager et al., 2012). The microtubule‐stabilizing and actin nucleation activities of Dia2 are known to be separate. However, if DIP inhibits the microtubule stabilizing activity of Dia2 is not clear. In prostate cancer cells, the loss of Dia2 promotes the formation of lung metastases after tail vein injection in immunocompromised mice (Hager et al., 2012). Interestingly, the loss of Dia2 also leads to hyperactivation of EGFR signaling, as the disruption of the microtubule lattice leads to the accumulation of EGFR in early endosomes (Hager et al., 2012). Dia2 is also negatively regulated by EGFR signaling, which may further activate EGFR signaling through a positive feedback loop (Figure 4) (Hager et al., 2012). These observations may help to explain the high level of Erk activity in prostate cancer cells that are not known to harbor activating mutations in this pathway. Thus, Dia1, Dia2, and DIP form the core of an actin regulatory network important for amoeboid migration.

### 1.5 Additional activities of the Diaphanous related formins

The mammalian DRF family is comprised of the Dia, DAAM, FMNL, and FHOD formins that are distinct in their GTPase binding specificity. The formin homology domain (FHOD) containing proteins, such as FHOD1, is known to bind ROCK1 (Hannemann et al., 2008). While ROCK1 activity is unaffected by this interaction, FHOD1 is activated by ROCK1 phosphorylation on three residues (S1131, S1137, and T1141) (Hannemann et al., 2008). The activation of FHOD1 by ROCK1 was found to promote blebbing in ovarian cancer cells (Hannemann et al., 2008). Moreover, the activation of FHOD1 by ROCK1 promoted invasion into hydrogels (Hannemann et al., 2008). The interaction of FHOD1 with ROCK1 was found to be dependent on Src activity (Hannemann et al., 2008). FHOD3 has been shown to drive filopodia formation, whereas its absence can induce blebbing (Paul et al., 2015). In contrast, blebbing induced by formin‐like 1 (FMNL1) is ROCK1 and Src independent. Importantly, the γ splice variant of FMNL1 contains an N‐terminal myristylation site that targets it to the PM (Han et al., 2009). Moreover, PM targeting effects the DAD of FMNL1, relieving autoinhibition (Han et al., 2009). In cells, this splice variant induces blebbing; thus, amoeboid migration may be regulated by alternative splicing of FMNL1. FMNL2 was also found to promote invasion into hydrogels downstream of RhoC, which is frequently upregulated in cancer (Kitzing et al., 2010). Notably, RhoC is highly similar in sequence to RhoA/B but differs slightly in a region important for effector interactions (Wheeler & Ridley, 2004). Thus, RhoA, B, and C may activate a unique complement of formins in amoeboid migrating cells.

Although disheveled associated activator of morphogenesis 1 (DAAM1) contains FH1 and FH2 domains, it has weak actin assembly activity. Due to its unique three‐dimensional structure, the surfaces for barbed end binding are partially occluded in DAAM1 (Lu et al., 2007). Instead, DAAM1 has been found to function as a signaling scaffold. More specifically, autoinhibition of DAAM1 is relieved by the binding of disheveled (Dvl) and not Rho GTPases (Liu et al., 2008). Upon activation by Dvl, DAAM1 recruits and maintains RhoA in an activate state through binding its GBD. In melanoma cells, the secreted Wnt ligands, 11 and 5B, activate RhoA, which requires DAAM1 (Rodriguez‐Hernandez et al., 2020). Consequently, Wnt11/5B promote amoeboid migration in melanoma. This work highlights the diverse mechanisms by which formins may regulate amoeboid migration.

## 2 CONCLUSION

The adoption of an amoeboid phenotype, characterized by the formation of blebs, drives metastasis in varied ways. In addition to migration, blebs have been found to be sites of heightened oncogenic signaling (Weems et al., 2023). In doing so, blebs help to confer resistance to anoikis, which is a hallmark of cancer (Weems et al., 2023). Additionally, increased self‐renewal or stemness is associated with an amoeboid phenotype (Rodriguez‐Hernandez et al., 2020). Thus, there is a strong rational for therapeutically targeting amoeboid cells in cancer. Based on the work reviewed here, the Diaphanous related formins are worthy of further scrutiny in animal models of cancer, as they have been shown to promote or suppress the amoeboid phenotype. It will also be essential to decipher the cell type‐specific activity of each formin. This includes immune cell types, as they also utilize amoeboid migration for tissue surveillance. The ideal therapy would only inhibit the amoeboid phenotype in cancer cells. How each formin promotes the amoeboid phenotype at the level of the cytoskeleton, that is, the architecture of the actin network, is also poorly understood. Recent advances in light microscopy are likely to reveal these changes at high resolution, whereas AFM will provide insight into how each of the formins affects the generation of intracellular pressure that would drive the formation of blebs. Thus, future work on the role of formins in regulating amoeboid migration in cancer and immune cells is likely to remain an important area of research for years to come.

## AUTHOR CONTRIBUTIONS

**N.K.**: Writing—Original Draft, Review & Editing. **J.S.L.**: Writing—Original Draft, Review & Editing, Funding acquisition.

## FUNDING INFORMATION

This work was supported by grants from the American Cancer Society (ACS; award no. RSG‐20‐019‐01—CCG) and the National Institutes of Health (NIH; award no. 1R35GM146588‐01) to J.S.L.

## CONFLICT OF INTEREST STATEMENT

The authors declare that they have no conflict of interest.

## Data Availability

Data sharing is not applicable to this article as no new data were created or analyzed in this study.
